# Protection of catalpol against triptolide-induced hepatotoxicity by inhibiting excessive autophagy via the PERK-ATF4-CHOP pathway

**DOI:** 10.7717/peerj.12759

**Published:** 2022-01-05

**Authors:** Linluo Zhang, Changqing Li, Ling Fu, Zhichao Yu, Gengrui Xu, Jie Zhou, Meiyu Shen, Zhe Feng, Huaxu Zhu, Tong Xie, Lingling Zhou, Xueping Zhou

**Affiliations:** 1 Department of First Clinical College, Nanjing University of Traditional Chinese Medicine, Nanjing City, Jiangsu, China; 2 Department of Second Clinical College, Nanjing University of Traditional Chinese Medicine, Nanjing City, Jiangsu, China; 3 Department of Pharmacy, Nanjing University of Traditional Chinese Medicine, Nanjing City, Jiangsu, China

**Keywords:** Hepatotoxicity, Autophagy, ERS, Triptolide, PERK, Catalpol

## Abstract

Catalpol significantly reduces triptolide-induced hepatotoxicity, which is closely related to autophagy. The aim of this study was to explore the unclear protective mechanism of catalpol against triptolide. The detoxification effect of catalpol on triptolide was investigated in HepaRG cell line. The detoxification effects were assessed by measuring cell viability, autophagy, and apoptosis, as well as the endoplasmic reticulum stress protein and mRNA expression levels. We found that 5–20 µg/L triptolide treatments increased the levels of alanine aminotransferase (ALT), aspartate aminotransferase (AST), and lactate dehydrogenase (LDH), as well as the expression of autophagy proteins including LC3 and Beclin1. The expression of P62 was downregulated and the production of autophagosomes was increased, as determined by transmission electron microscope and monodansylcadaverine staining. In contrast, 40 µg/L catalpol reversed these triptolide-induced changes in the liver function index, autophagy level, and apoptotic protein expression, including Cleaved-caspase3 and Cleaved-caspase9 by inhibiting excessive autophagy. Simultaneously, catalpol reversed endoplasmic reticulum stress, including the expression of PERK, which regulates autophagy. Moreover, we used the PERK inhibitor GSK2656157 to prove that the PERK-ATF4-CHOP pathway of the unfolded protein response is an important pathway that could induce autophagy. Catalpol inhibited excessive autophagy by suppressing the PERK pathway. Altogether, catalpol protects against triptolide-induced hepatotoxicity by inhibiting excessive autophagy via the PERK-ATF4-CHOP pathway. The results of this study are beneficial to clarify the detoxification mechanism of catalpol against triptolide-induced hepatotoxicity and to promote the application of triptolide.

## Introduction

*Tripterygium wilfordii Hook F.* (TW), a traditional Chinese medicinal herb, has been clinically and widely used to treat various tumors and inflammatory and autoimmune diseases because of its remarkable therapeutic effects. However, severe adverse reactions, including hepatotoxicity, restrict the practical applications of TW (Li, Jiang & Zhang, 2014). Triptolide (TP), one of the main effective and toxic ingredients of TW (Fu et al., 2020), may significantly reduce liver injury once its therapeutic effects are realized (Wang et al., 2013). It is one of the focal points that clarifying the main mechanism of TP-induced hepatotoxicity and reducing/preventing its hepatotoxicity while guaranteeing its therapeutic effect.

Drug compatibility is an important method to reduce toxicity. In the permanent clinical practice of traditional Chinese medicine (TCM), TW is frequently used with a formula that contains a variety of Chinese herbs to obtain synergistic effects and/or alleviate possible adverse reactions (Feng et al., 2019), which is called the compatibility of TCM. For example, the proficient National Chinese Medicine Master Zhongying Zhou’s prescription Qingluotongbi Formula (QLT) is such a compound which includes TW. It has been used to treat rheumatoid arthritis of Yin Deficiency and Collateral Heat Syndrome for decades, and proved to be effective (Liu et al., 2015) and non-hepatotoxic. In our previous studies, we found that *Rehmannia glutinosa* (RG), a component of QLT, reduced TW-induced hepatotoxicity (Zhang et al., 2016), and catalpol (CAT), the main active ingredient of RG, remarkably protected against TP-induced hepatotoxicity (Zhou et al., 2018).

Autophagy is a highly evolutionarily conserved protective mechanism for maintaining homeostasis of the intracellular environment, and is a well-recognized mode of TP-induced hepatotoxicity (Huo et al., 2019; Yuan et al., 2019). Autophagy intimately connects with endoplasmic reticulum stress (ERS) (Levine & Kroemer, 2008), which may maintain cell survival and also cause apoptosis or autophagic cell death. A recent study showed that drp1-associated mitochondrial dysfunction and mitochondrial hyper autophagy are important mechanisms in TP-induced liver injury (Hasnat et al., 2019). CAT can inhibit ERS and regulate autophagy to reduce toxicity and resist inflammation (Bi et al., 2020; Liu et al., 2018). Some studies have shown that CAT decreases the upregulation of autophagy to protect against 2,3,7,8-tetrachlorodibenzo-p-dioxin-induced damage (Choi et al., 2019). However, the exact mechanism by which CAT alleviates TP-induced hepatotoxicity, particularly autophagy, remains unclear and requires further studying.

ER is responsible for the quality control of bioactive proteins, such as synthesis, folding, and post-translational modification, which regulates autophagy in TP-induced hepatotoxicity and plays a key role in cell function (Hwang & Qi, 2018). Reactive oxygen species (ROS) and severe ERS may activate multiple signaling pathways that lead to autophagic cell death, and the integration of these responses is critical to the pathogenesis of various diseases, including drug-induced liver injury (Song et al., 2017). The significant role of oxidative stress caused by ROS in TP-induced damage has also been observed (Chan et al., 2017; You et al., 2018). A previous study showed that TP-induced oxidative stress in human hepatic cells and led to cytotoxicity (Feng et al., 2019). Under ERS, cells initiate an unfolded protein response (UPR) to maintain a homeostatic balance. However, under long-term or excessive ERS, UPR is not sufficient to restore ER homeostasis and eventually leads to cell death (Iurlaro & Muñoz Pinedo, 2016; Wang et al., 2018). Among the three important arms of UPR induced by ERS, the PERK pathway plays a key role in regulating autophagy. Studies have shown that, through the PERK-ATF4-CHOP pathway, classical swine fever virus infection induced ERS and complete autophagy both *in vivo* and *in vitro* (He et al., 2017; Zhu et al., 2019). However, whether ERS exacerbates abnormal autophagy through the PERK pathway and whether the suppression of this pathway mediates the protective effection of CAT against TP-induced hepatotoxicity requires further study.

Therefore, the aim of this study was to explore the action of autophagy and the relationship between autophagy and apoptosis, and autophagy and ERS in TP-induced hepatotoxicity. We also determined whether the PERK pathway plays an important regulatory role in autophagy. The results may elucidate whether the protective mechanism of CAT in alleviating TP-induced toxicity is related to the PERK-ATF4-CHOP pathway.

### Material and methods

### Cell cultures and reagents

HepaRG cells, a preferable cell model for drug-induced liver injury (DILI) (Wu et al., 2016), were used for *in vitro* experiments. The HepaRG cell line was purchased from Beina Chuanglian Biotechnology (Beijing, China). The cells were cultured in RPMI 1640 medium containing 10% fetal bovine serum (FBS) and 1% antibiotics, and then incubated in a cell incubator containing 5% CO_2_ at 37 °C.

CAT (purity ≥ 98.0%) and TP (purity ≥ 98.0%) were purchased from Yuanye Biotechnology (Shanghai, China). The stock solution of TP (20 mg/mL) was dissolved in dimethyl sulfoxide (DMSO) and diluted with basal medium to various concentrations (0, 5, 10, and 20 µg/L). CAT (20 mg/mL) dissolved in DMSO was diluted to various concentrations (0, 0.4, 4, 40, 400, 4,000, and 40,000 µg/L) with RPMI 1640.

### Cell viability assay using cell counting kit 8 (CCK8)

Cell proliferation was assessed using the CCK8 (Beyotime, Shanghai, China) assay. To detect cell toxicity of TP, HepaRG cells (5 × 10^4^ cells/mL) were plated into 96-well plates for 24 h, and then treated with different doses of TP for 12, 24, 36, and 48 h. To detect the protective effect of CAT, the cells were pretreated with CAT (0, 0.008, 0.04, 0.2, and 1 µg/L) for 12 h, and then incubated with TP (20 µg/L) for 24 h. After incubating with 10 µL CCK8 solution for 3 h, the optical density value was determined at an excitation wavelength of 450 nm using a microplate reader (TECAN, Switzerland).

In addition, to evaluate the hepatotoxicity, the supernatant of cells incubated with TP/CAT was used to detect the total alanine aminotransferase (ALT), aspartate aminotransferase (AST), and lactate dehydrogenase (LDH) levels using detection kits (Jiancheng Bioengineering, Nanjing, China).

### Detection of autophagosomes by transmission electron microscope (TEM)

After incubation, the cells (3 × 10^5^ cells/mL) were treated with electron microscope fixative, fixed for 2 h at 25 °C, cut into small pieces (1 mm^3^), fixed, dehydrated, penetrated and embedded, sliced (1–2 µm) and axial lead stained. The specimens were observed by TEM.

To demonstrate the relationship between autophagy and apoptosis, the autophagy inhibitor 3-methyladenine (3-MA) (2.5 mM) and agonist rapamycin (RAPA) (50 nM) were used. Furthermore, the ERS inhibitor 4-phenylbutyric acid (4-PBA) (500 nM) and PERK inhibitor GSK2656157 (1 µM) (Bastida-Ruiz et al., 2019; Wang et al., 2020) were also used to study the relationship between PERK and autophagy.

### Autophagosome detection by fluorescence microscope

Acidic autophagic vacuoles were measured using monodansylcadaverine (MDC) staining (Veeran et al., 2017) with an autophagy detection kit (KeyGEN, Nanjing, China). The cells (1 × 10^5^ cells/mL) were plated in 24-well plates and incubated with different concentrations of TP/CAT for 24 h at 37 °C. The cells were treated according to the manufacturer’s instructions and observed at an excitation wavelength of 355 nm using a fluorescence microscope (ZEISS, Thuringia, Germany).

### Flow cytometry for apoptosis

The apoptotic ratio was detected using an Annexin V-FITC/PI Apoptosis Detection kit (Vazyme, Nanjing, China) and measured by flow cytometry (Beckman Coulter, CA, USA). Early apoptotic cells are single positive for Annexin V-FITC, and late apoptotic cells are double positive for Annexin V-FITC and PI (Chen et al., 2008). As triptolide-induced apoptosis involves early and late apoptosis (You et al., 2018), the apoptosis rate was calculated as the sum of the ratios of early and late apoptosis. Data were analyzed using FlowJo V 10.

### Measurement of reactive oxygen species (ROS)

The content of intracellular ROS was measured using the DCFH-DA fluorescent dye (Wang et al., 2017) with the ROS Assay Kit (Beyotime, Shanghai, China). After incubation, the cells were treated with 10 µM DCFH-DA reagent and incubated in the dark at 37 °C for 20 min. Fluorescence was measured by flow cytometry at an excitation and emission wavelengths of 488 nm and 530 nm.

### Western blotting

HepaRG cells (4 × 10^5^ cells/mL) were seeded in six-well plates overnight and then treated with different concentrations of TP/CAT for 24 h. The cells were collected and lysed using RIPA buffer (Beyotime, Shanghai, China) on ice for 30 min. The total protein concentration was detected using a BCA protein assay kit (Beyotime, Shanghai, China). The protein lysates (30 µg) were separated on 12% sodium dodecyl sulfate-polyacrylamide gel electrophoresis (SDS-PAGE) and transferred onto a polyvinylidene fluoride (PVDF) membrane (Millipore, Darmstadt, Germany). The membranes were incubated overnight at 4 °C with the following primary antibodies: p-PERK (CST, Danvers, MA, USA), LC3, SQSTM1/P62, Beclin1, GRP78/BIP, PERK, IRE1, ATF6, ATF4, CHOP, BCL2, Cleaved-caspase3, Cleaved-caspase9, and GAPDH (Proteintech, Chicago, IL, USA). After incubating with the secondary antibodies at room temperature for 1 h, the protein membranes were detected using the ECL system (Bio-Rad, Hercules, CA, USA).

### Quantitative real-time PCR (qRT-PCR)

The total RNA from cells was extracted, and the mRNA expression levels of *LC3*, *P62*, Beclin1, *BAX*, *BCL2*, *GRP78*, and *GAPDH* was detected using qRT-PCR. The primer pairs are listed in Table 1. Relative expression was calculated as fold changes compared with the control gene GAPDH using the 2^−^^△△^CT method.

**Table 1:** Primers in qRT-PCR.

Target gene		Sequence of primers (5′ to 3′)
*LC3*	Forward:	CCTGGACAAGACCAAGTTTTTG
	Reverse:	GTAGACCATATAGAGGAAGCCG
*P62*	Forward:	CAGGCGCACTACCGCGATG
	Reverse:	ACACAAGTCGTAGTCTGGGCAGAC
*Beclin1*	Forward:	CTGAAACTGGACACGAGCTTCAAG
	Reverse:	TGTGGTAAGTAATGGAGCTGTGAGTT
*BAX*	Forward:	CCCGAGAGGTCTTTTTCCGAG
	Reverse:	CCAGCCCATGATGGTTCTGAG
*BCL2*	Forward:	GGTGGGGTCATGTGTGTGG
	Reverse:	CGGTTCAGGTACTCAGTCATCC
*GRP78*	Forward:	GGAGCGTCTGATTGGCGATGC
	Reverse:	CATTCCAAGTGCGTCCGATGAGG
*GAPDH*	Forward:	CACCATCTTCCAGGAGCGAG
	Reverse:	AAATGAGCCCCAGCCTTCTC

### Statistical analysis

Data were expressed as mean ± standard deviation (SD) and were analyzed using GraphPad Prism 8.0.2. One-way ANOVA followed by Dunnett post-hoc test and *t*-test were used to compare the means of multiple or two groups, respectively. Difference was considered significant at *p* < 0.05.

## Results

### CAT defends from TP-induced hepatotoxicity

**Figure 1:**
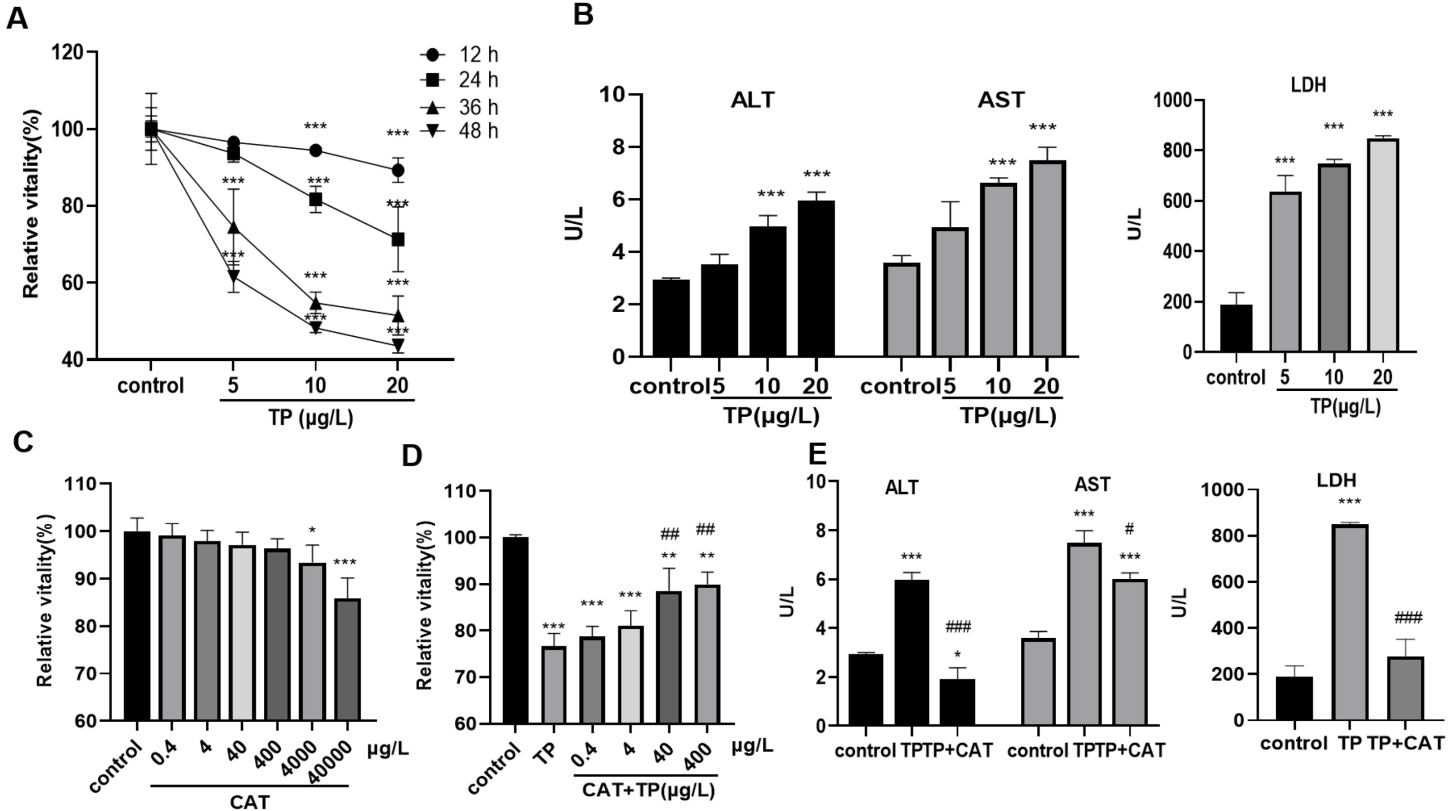
CAT defends from TP-induced hepatotoxicity. (A) Relative viability of cells at different time points and doses of TP using CCK8. (B) The levels of ALT, AST, and LDH (U/L) in cells incubated with TP. (C) Relative viability of cells exposed to different doses of CAT. (D) Relative viability of HepaRG cells treated with CAT and TP. (E) CAT (40 µ g/L) reversed the increase in ALT, AST, and LDH levels (U/L) caused by TP. Data are presented as mean ± SD (*n* = 3). *P < 0.05, **P < 0.01, ***P < 0.001 *vs.* control. #*P* < 0.05, ##*P* < 0.01, ###*P* < 0.001 *vs.* TP.

The results of the CCK8 assay showed that, 5–20 µg/L TP decreased the relative activity of cells for 12, 24, 36, and 48 h in a time- and dose-dependent manner (Fig. 1A). Cell toxicity was moderate under 20 µg/L TP treatment for 24 h, and the concentration was used in the subsequent experiments. Simultaneously, the levels of ALT, AST, and LDH in the culture medium increased (Fig. 1B). Using the CCK8 assay, the relative safe dose of CAT was determined to be 0.4–400 µg/L (Fig. 1C). The protective effect of CAT was the most obvious when CAT was used at 40 and 400 µg/L. Based on this, the actual dose of CAT in the subsequent experiments was set to 40 µg/L. CAT reversed the TP-induced decrease in cell viability (Fig. 1D) and the levels of AST, ALT, and LDH (Fig. 1E). The results indicated that CAT could defend from TP-induced hepatotoxicity.

### TP-induced overactivation of autophagy is inhibited by CAT

**Figure 2:**
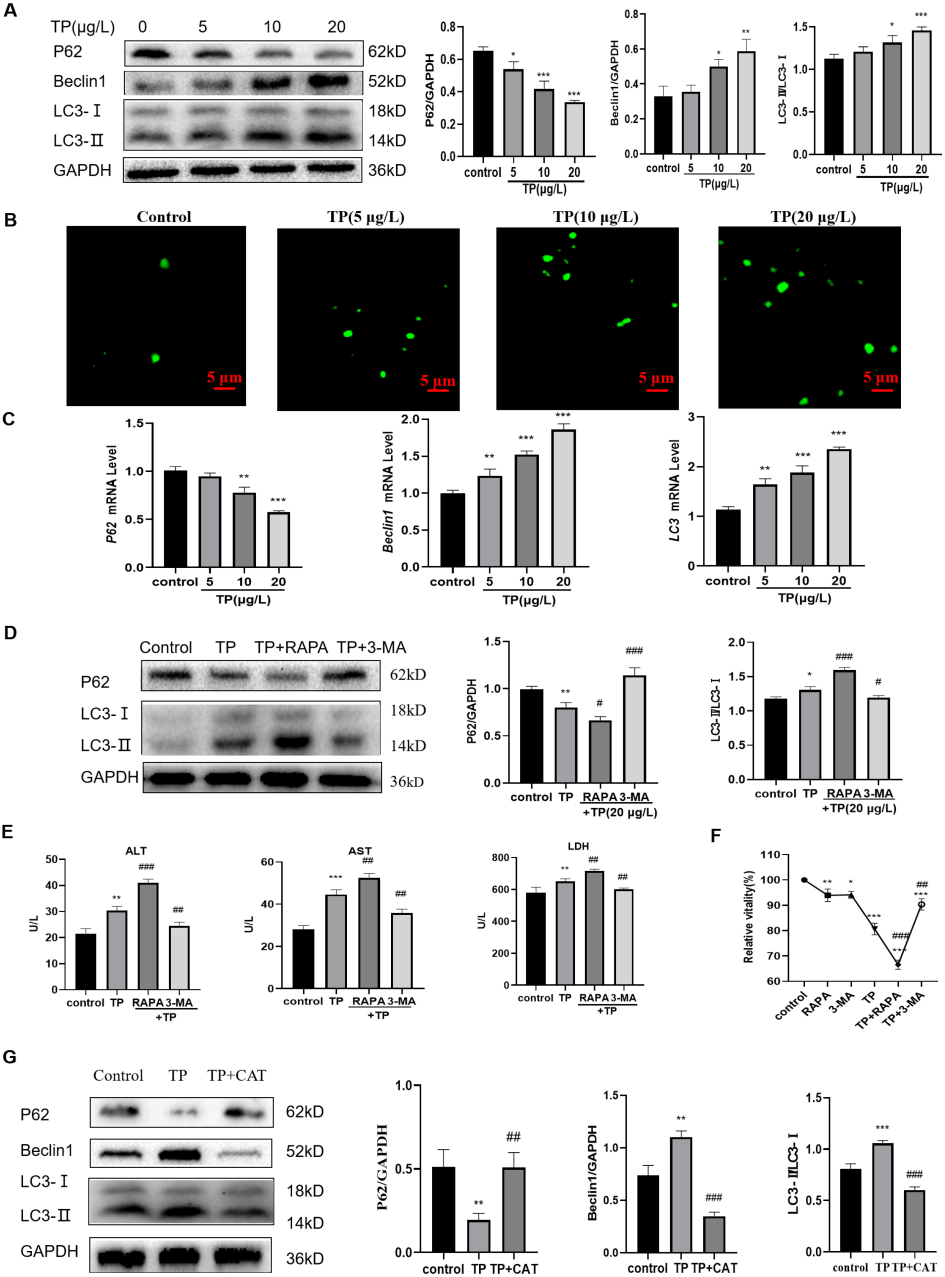
TP induces excessive autophagy, which is inhibited by CAT. (A) By western blotting, TP-induced changes in Beclin1, P62, and LC3 expression levels were determined. (B) MDC staining revealed that the change in autophagosomes caused by TP. Green vesicles represent autophagosomes (200× magnification, Scale bar: 50 µm). (C) The changes in mRNA levels of *LC3*, *P62*, and *Beclin1* at different doses of TP. (D) RAPA (50 nM) and 3-MA (2.5 mM) used in combination with TP caused changes in the levels of autophagy proteins, LC3 and P62. (E) RAPA and 3-MA used in combination with TP caused changes in the levels of ALT, AST, and LDH (U/L). (F) RAPA and 3-MA induced changes in the relative cell viability caused by TP. (G) CAT reversed the protein expression of LC3, P62, and Beclin1 caused by TP. Data are presented as mean ± SD (*n* = 3). *P < 0.05, **P < 0.01, ***P < 0.001 *vs.* control. #*P* < 0.05, ##*P* < 0.01, ###*P* < 0.001 *vs.* TP.

Excessive autophagy is one of the important mechanisms by which TP induces hepatotoxicity (Huo et al., 2019); thus, we focused on investigating autophagy. At 5–20 µg/L TP, the expression of the autophagy proteins, LC3 and Beclin1, was upregulated, whereas that of P62 was downregulated compared with those in the control group (Fig. 2A). By MDC staining, fluorescence microscopy suggested that the production of autophagosomes also increased as the TP dosage increased (Fig. 2B). Simultaneously, the corresponding mRNA levels of *LC3*, *Beclin1*, and *P62* also showed tendencies with those of the consistent proteins (Fig. 2C).

When RAPA (50 nM), an effective and specific mTOR inhibitor, was used to further activate autophagy, the expression level of LC3 was upregulated and that of P62 was downregulated (Fig. 2D). Meanwhile, the levels of ALT, AST, and LDH further increased (Fig. 2E). When 3-MA (2.5 mM), a pan class III PI3K inhibitor, was used to inhibit autophagy, the expression of LC3 was downregulated and that of P62 was upregulated (Fig. 2D), and the elevated levels of ALT, AST, and LDH levels were reversed (Fig. 2E). In addition, cell viability also changed in line with liver function indexes when 3-MA, RAPA, and TP were used in combination (Fig. 2F). These results indicated that excessive autophagy is one of the main mechanisms of TP-induced liver cell damage.

As expected, CAT downregulated LC3 and Beclin1, and upregulated P62 (Fig. 2G). Simultaneously, elevated levels of ALT, AST, and LDH were reversed by CAT (Fig. 1E), and cell viability was also improved (Fig. 1D)

### TP-induced apoptosis is restrained by CAT *via* hyperautophagy repression

**Figure 3:**
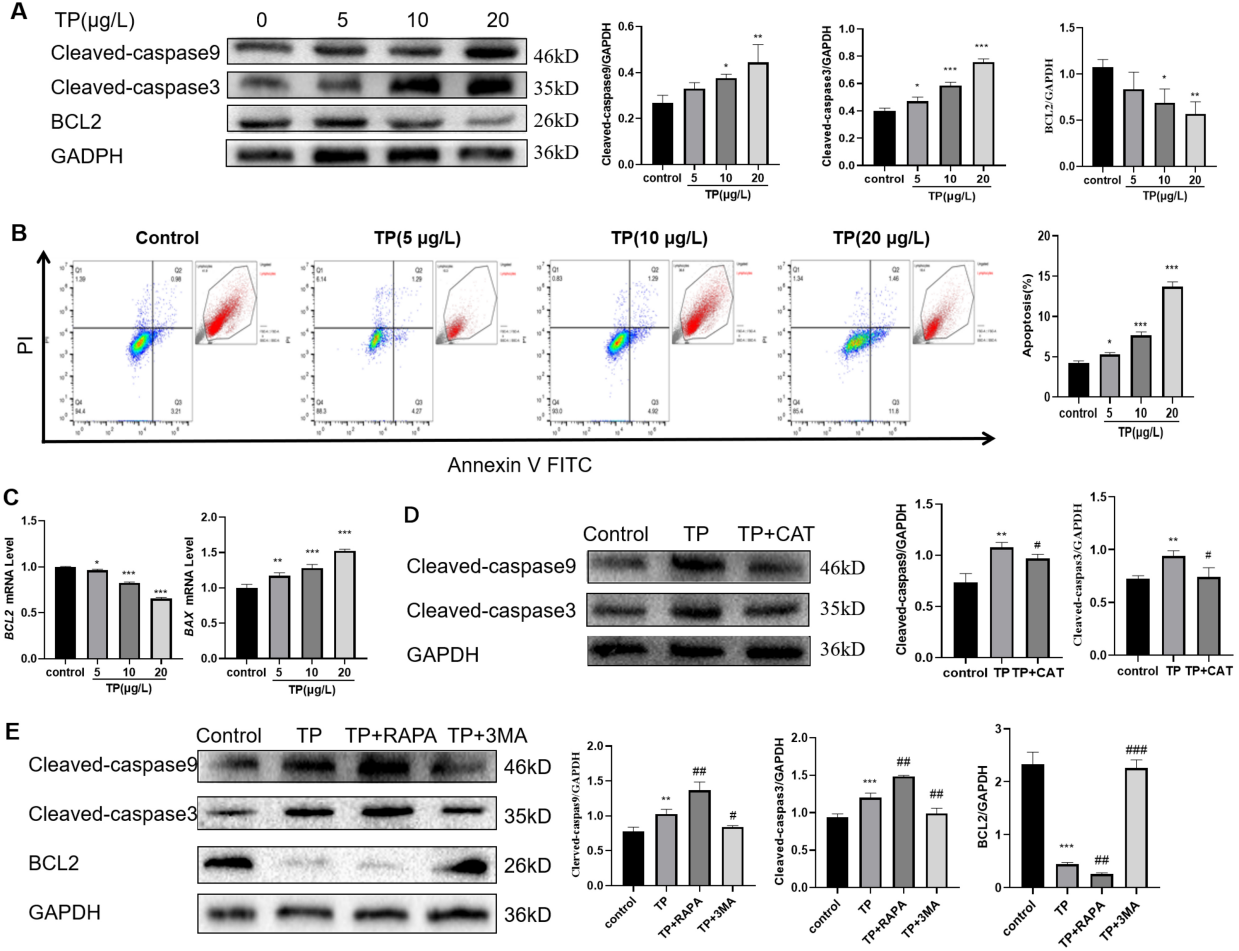
TP-induced apoptosis is restrained by CAT *via* hyperautophagy repression. (A) Western blotting revealed that TP induced expression changes in Cleaved-caspase3, Cleaved-caspase9, and BCL2. (B) The changes in the apoptotic ratio were measured by flow cytometry. (C) mRNA changes in *BAX* and *BCL2* at different doses of TP. (D) CAT reversed the TP-induced increase in Cleaved-caspase3 and Cleaved-caspase9. (E) RAPA (50 nM) and 3-MA (2.5 mM) used with TP induced changes in apoptotic proteins Cleaved-caspase3, Cleaved-caspase9, and BCL2. Data are presented as mean ± SD (*n* = 3). *P < 0.05, **P < 0.01, ***P < 0.001 *vs.* control. #*P* < 0.05, ##*P* < 0.01, ###*P* < 0.001 *vs.* TP.

Considering the role of apoptosis in TP-induced liver injury, we also detected the level of apoptosis. The results showed that with the increase in TP exposure, the apoptotic protein levels of Cleaved-caspase3 and Cleaved-caspase9 increased, and the anti-apoptotic protein level of BCL2 decreased (Fig. 3A). Flow cytometry revealed that 5–20 µg/L TP increased the number of apoptotic cells (Fig. 3B). The mRNA level of *BAX* increased, whereas that of *BCL2* decreased (Fig. 3C). CAT reversed the protein expression levels of Cleaved-caspase3 and Cleaved-caspase9 (Fig. 3D).

To investigate the relationship between autophagy and apoptosis in TP-induced hepatotoxicity, we further used RAPA and 3-MA. When RAPA (50 nM) was used to upregulate autophagy, the level of Cleaved-caspase3 and Cleaved-caspase9 was upregulated and that of BCL2 was downregulated; when 3-MA (2.5 mM) was used to downregulate autophagy, the level of Cleaved-caspase3 and Cleaved-caspase9 was downregulated and that of BCL2 was upregulated (Fig. 3E). Thus, excessive autophagy induced apoptosis in TP-induced live cell damage.

In summary, CAT could restrain apoptosis by inhibiting excessive autophagy in TP-induced liver cell damage.

### CAT protects against TP-induced extreme autophagy by inhibiting ERS

**Figure 4:**
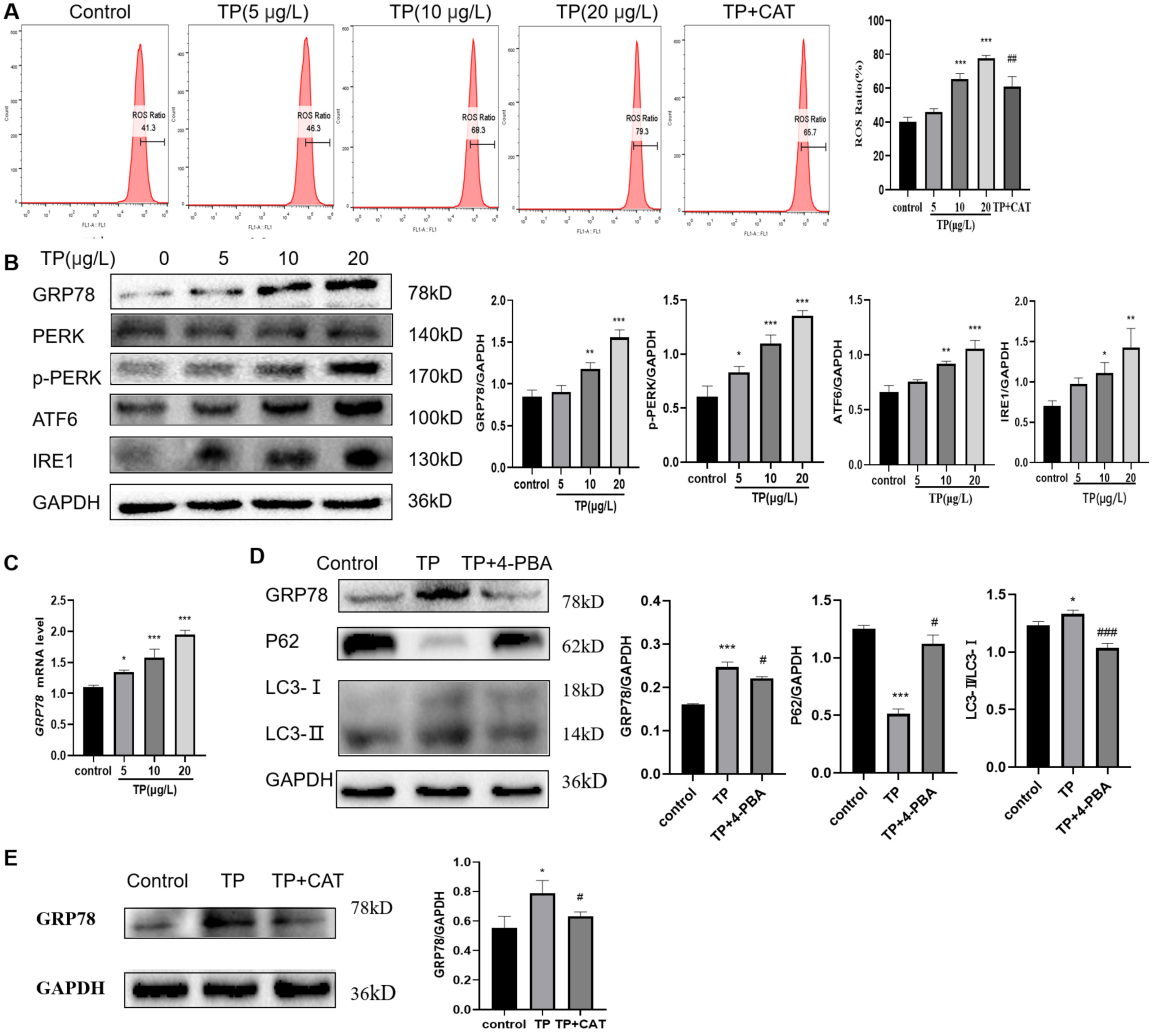
CAT reverses the increase in the ROS ratio and ERS caused by TP. (A) Flow cytometry analysis of TP-induced increase in the ROS ratio, whereas CAT reduced the ROS ratio. (B) By western blotting, the protein levels of GRP78, IRE1, ATF6, and PERK were upregulated by TP. (C) The qRT-PCR analysis revealed that TP elevated the mRNA level of *GRP78*. (D) 4-PBA caused protein expression changes in GRP78 and autophagy (LC3 and P62). (E) CAT reduced the protein expression of GRP78 caused by TP. Data are presented as mean ± SD (*n* = 3). *P < 0.05, **P < 0.01, *** *P* < 0.001 *vs.* control. #*P* < 0.05, ##*P* < 0.01, ###*P* < 0.001 *vs.* TP.

TP caused an increase in the ROS ratio as determined flow cytometry (Fig. 4A). Meanwhile, western blotting and qRT-PCR analysis indicated that GRP78, IRE1, ATF6, and PERK, the markers of ERS, were upregulated at the protein (Fig. 4B) and mRNA levels (Fig. 4C). To probe the relationship between autophagy and ERS in TP-induced hepatotoxicity, we used 4-PBA, an ERS inhibitor. When 4-PBA (500 nM) was used along with TP to downregulate ERS, the autophagy marker protein LC3 was downregulated and P62 was upregulated (Fig. 4D).

As expected, CAT decreased the ROS generation ratio (Fig. 4A) and downregulated the protein level of GRP78, which was elevated by TP (Fig. 4E); whereas, the protein level of LC3 and Beclin1 was decreased and that of P62 was increased (Fig. 2G).

In summary, ERS is an important factor in regulating autophagy, and CAT can inhibit excessive autophagy caused by TP by restraining ERS.

### CAT restrains fulsome activation of autophagy by the PERK-ATF4- CHOP pathway

**Figure 5:**
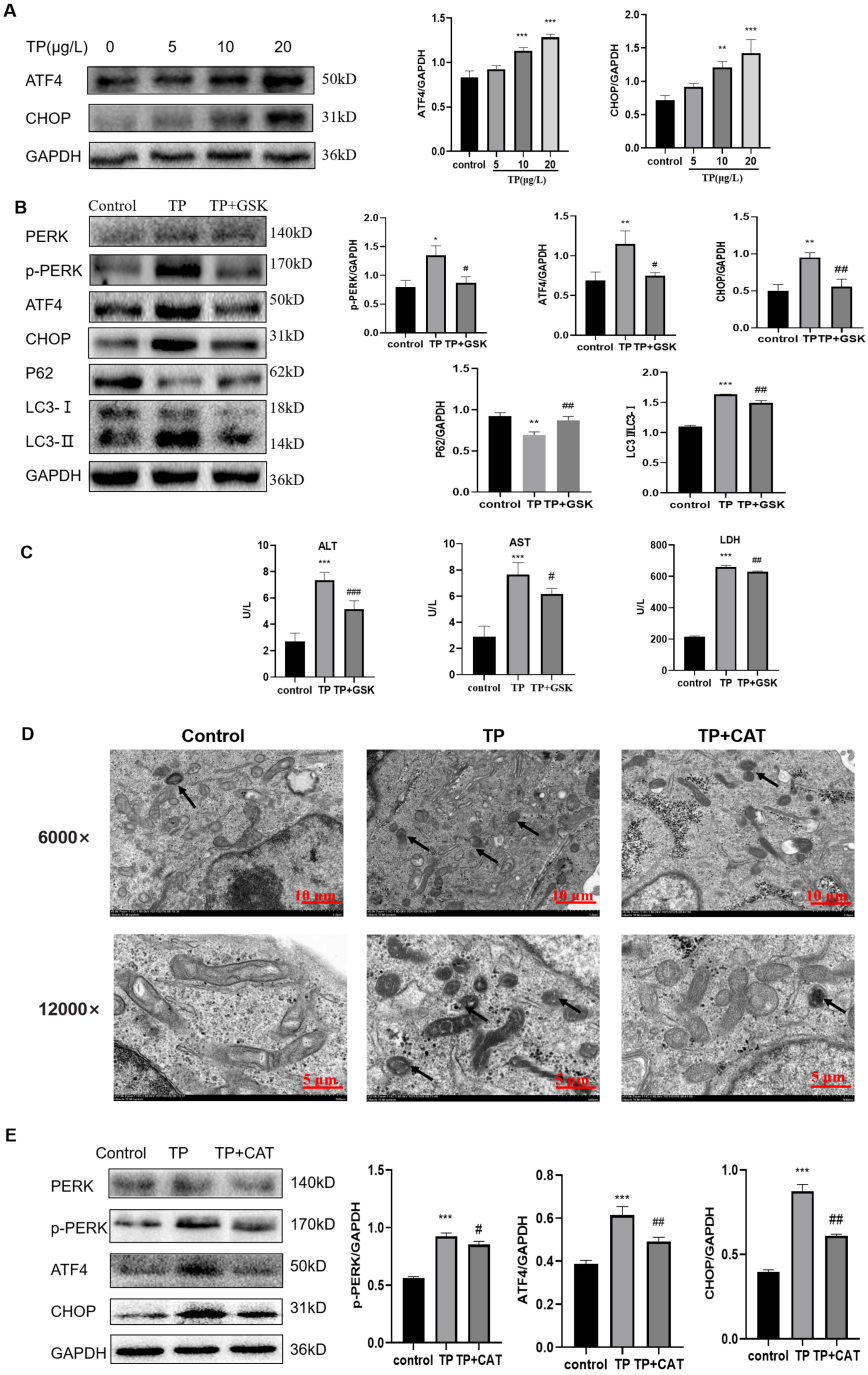
CAT restrains excessive autophagy by inhibiting the PERK-ATF4-CHOP pathway. (A) By western blotting, the effects of TP on the expression of p-PERK, ATF4, and CHOP were determined. (B) GSK inhibited the autophagy protein LC3 and upregulated P62 by lowering PERK. (C) GSK reversed the increase in the levels of ALT, AST, and LDH (U/L). (D) Changes in autophagolysosomes under TEM. Black arrow: autophagolysosome. (6,000×/12,000× magnification, Scale bar: 100/50 µm). (E) CAT reversed the TP-induced increased in the levels of p-PERK, ATF4, and CHOP. Data are presented as mean ± SD (*n* = 3). *P < 0.05, **P < 0.01, ***P < 0.001 *vs.* control. #*P* < 0.05, ##*P* < 0.01, ###*P* < 0.001 *vs.* TP.

Western blotting revealed that the expression of p-PERK, ATF4, and CHOP, three key proteins in the PERK pathway, was upregulated by TP in a dose-dependent manner (Fig. 5A) and the autophagy flux also increased (Fig. 2B).

To explore whether the PERK-ATF4-CHOP pathway is a key regulator of TP-induced hype autophagy, we further applied GSK2656157, a selective ATP-competitive PERK inhibitor. When GSK2656157 (1 µM) inhibited the PERK pathway of ERS, LC3 was downregulated and P62 was upregulated (Fig. 5B), and simultaneously, the ALT, AST, and LDH levels also decreased (Fig. 5C). Thus, the PERK-ATF4-CHOP pathway plays an important role in the regulation of fulsome autophagy.

As envisioned, CAT reversed TP-induced upregulation of autophagic flux under TEM (Fig. 5D), and the changes in GRP78 (Fig. 4E), LC3, Beclin1, and P62 (Fig. 2G) by inhibiting the PERK-ATF4-CHOP pathway (Fig. 5E). Simultaneously, elevated ALT, AST, and LDH levels were also reversed (Fig. 1E).

Overall, CAT inhibited ERS induced by TP through suppressing the PERK-ATF4-CHOP pathway to restrain excessive autophagy to protect liver cells.

## Discussion

The application of TP, a main active and toxic ingredient extracted from TW, is limited owing to its potential liver toxicity (Huang et al., 2021). CAT, an active ingredient of RG, has several activities, including antioxidant, liver-protection, anti-inflammatory, anti-ERS, anti-aging, and anti-tumor effects (Bi et al., 2020; Liu et al., 2017; Zhang, Chen & Li, 2019). It has been shown that CAT can effectively reduce the liver toxicity of TP (Feng et al., 2019; Fu et al., 2020). However, the clear protection mechanism of CAT against TP-induced hepatotoxicity is still not very explicit.

Macroautophagy (abbreviated as autophagy), a type II cell programmed death mechanism, can protect cells by removing damaged organelles and proteins (Klionsky & Emr, 2000). The key steps in autophagy include initiation, formation of isolation membrane or phagophore, vesicle elongation extending into autophagosomes, autophagosome and lysosome fusion with autophagolysosomes, and degradation of autophagolysosomes (Mizushima, Yoshimori & Ohsumi, 2011). Among them, the formation of autophagosomes is a marker of autophagy. Broadly, a moderate level of autophagy removes damaged organelles and macromolecular proteins, thereby inhibiting apoptosis to protect cells. However, excessive autophagy induces apoptosis or autophagic cell death (Nowikovsky & Bergmann, 2017). Abnormal regulation of autophagy is related to various liver diseases including drug-induced liver injury, and its adjustment is considered as a potential new treatment strategy (Allaire et al., 2019). Conversion of LC3-I to LC3-II is an indicator of autophagosome formation; while P62 is a substrate for autophagolysosome degradation and Beclin1 is necessary to regulate autophagy. In this study, with the increase in TP (5–20 µg/L) exposure, the protein level of the autophagy markers LC3 and Beclin1 increased and that of P62 decreased, which indicated that the autophagy flux increased. Considering that autophagosome changes under TEM are a criterion to detect autophagy, the changes in autophagosomes in HepaRG cells were observed by TEM and fluorescence microscope.

To further verify the role of autophagy in hepatotoxicity by TP, we used the autophagy agonist RAPA and inhibitor 3-MA. When autophagy was further activated by RAPA, cell viability was further reduced and liver function index was further aggravated. However, when autophagy was inhibited by 3-MA, cell viability was further increased, and liver function index was reversed. As the role of apoptosis in TP-induced hepatotoxicity (You et al., 2018) and the relationship between autophagy and apoptosis is intricate, we explored their connection. When RAPA was used to further induce autophagy, the protein level of Cleaved-caspase3 and Cleaved-caspase9 was upregulated, and simultaneously, the level of anti-apoptotic protein BCL2 was downregulated. When 3-MA was used to inhibit autophagy, the expression of Cleaved-caspase3 and Cleaved-caspase9 was downregulated and that of BCL2 was upregulated. Thus, autophagy induced by TP (20 µg/L) promoted apoptosis. As expected, CAT inhibited the expression of autophagic and apoptotic proteins (LC3, P62, Beclin1, Cleaved-caspase3, and Cleaved-caspase9) induced by TP to reduce liver function indicators.

The ER is the key to regulating proteins involved in synthesis, folding, transport, and degradation. As a crucial factor in regulating intracellular environmental homeostasis, the ER is sensitive to oxidative stress and plays a vital regulatory role in oxidative stress-induced injury. An increase in ROS induced by physical, chemical, or drug irritation is the main reason for ERS. ERS through UPR promotes cell survival or death if ERS is chronic or severe (Wang & Kaufman, 2016). This study showed that TP caused an increase in the intracellular ROS ratio and the marker of ERS glucose regulating protein 78 (GRP78) after TP exposure. However, CAT reduced ROS production and downregulated GRP78 expression, thus inhibiting ERS. Generally, under ERS, cells initiate UPR through three pathways: protein kinase R-like ER kinase (PERK), inositol requiring enzyme 1 (IRE1), and activating transcription factor-6 (ATF-6) to reduce damage. Among these, the PERK signaling is the preferred activation pathway induced by ERS (Fung, Torres & Liu, 2015). Through the phosphorylation of the α-subunit of eukaryotic initiation factor 2 (eIF2α), phosphorylated PERK (p-PERK) activates its downstream activating transcription factor 4 (ATF4), which can regulate the expression of multiple autophagy-related genes, including *LC3*, *ATG5*, *ATG7*, and *Beclin1* (Rzymski et al., 2009). C/EBP homologous protein (CHOP) is a non-endoplasmic reticulum-localized transcription factor induced by ERS, which forms heterodimers with ATF4 to regulate UPR, autophagy and mRNA translation by the related genes (Han et al., 2013). A study (Zhou et al., 2019) showed that sorafenib induced ERS and caused autophagy, which may be regulated by the PERK-ATF4-Beclin1 pathway. In our study, with the increase in TP concentration, the expression of GRP78, IRE1, ATF6, and PERK was upregulated; the expressions of LC3 and Beclin1 was also upregulated. As a reverse verification, when 4-PBA was used to downregulate ERS, the autophagy level was also downregulated. Thus, ERS exacerbates excessive autophagy in TP-induced toxicity.

In addition, after treatment with TP, the protein levels of p-PERK, ATF4, and CHOP in the PERK pathway were upregulated, and autophagy flux and liver function indexes were also upregulated. To further verify that excessive autophagy regulated by ERS in TP-induced damage was through the PERK-ATF4-CHOP pathway, we used the PERK inhibitor GSK2656157. When GSK2656157 was used to inhibit the protein expression of PERK, the levels of p-PERK, ATF4, and CHOP were downregulated, autophagy flux was also downregulated, and liver function indexes and relative cell viability were reversed. Similar to GSK2656157, CAT downregulated excessive autophagy by restraining the PERK-ATF4-CHOP pathway to reduce TP-induced liver cell damage.

## Conclusions

In summary, our study proved that TP-induced hepatotoxicity is closely related to excessive autophagy indued by ERS, mainly *via* the PERK-ATF4-CHOP pathway. CAT could reduce excessive autophagy by inhibiting ERS through the PERK-ATF4-CHOP pathway to reverse TP-induced damage. However, whether the IRE1 and ATF6 pathways of UPR activated by ERS are also involved in the regulation of autophagy in TP-induced hepatotoxicity remains to be further studied.

## Supplemental Information

10.7717/peerj.12759/supp-1 Supplemental Information 1 Results of CCK8 (TP treated for 12 h, 24 h, 36 h, and 48 h, and TP and CAT) and liver function (ALT, AST, LDH)

10.7717/peerj.12759/supp-2 Supplemental Information 2 Results of Western Blotting treated TP,TP and CAT (LC3, beclin1, P62, PERK, ATF4, CHOP, and GRP78)

10.7717/peerj.12759/supp-3 Supplemental Information 3 Results of Western Blotting and q-PCR treated TP, TP and CAT (LC3, beclin1, P62, PERK, ATF4, CHOP, and GRP78)

10.7717/peerj.12759/supp-4 Supplemental Information 4 Flow cytometry (Apoptosis) control: 5, 10, 20 ug/L

10.7717/peerj.12759/supp-5 Supplemental Information 5 Flow cytometry (ros) control: 5, 10, 20 ug/L

10.7717/peerj.12759/supp-6 Supplemental Information 6 Autophagy by Transmission electron microscopy Magnification 6,000 times

10.7717/peerj.12759/supp-7 Supplemental Information 7 Transmission electron microscopy Magnification 12,000 times

10.7717/peerj.12759/supp-8 Supplemental Information 8 Autophagy by Transmission electron microscopy Magnification 6,000 times

10.7717/peerj.12759/supp-9 Supplemental Information 9 Autophagy by Transmission electron microscopy Magnification 12,000 times

10.7717/peerj.12759/supp-10 Supplemental Information 10 Autophagy by Transmission electron microscopy Magnification 6,000 times

10.7717/peerj.12759/supp-11 Supplemental Information 11 Autophagy by Transmission electron microscopy Magnification 12,000 times

## Additional Information and Declarations

### Data Availability

The following information was supplied regarding data availability: The raw data is available in the Supplemental Files.
