# Supplementary figures and images for: Protection of catalpol against triptolide-induced hepatotoxicity by inhibiting excessive autophagy via the PERK-ATF4-CHOP pathway
Source: PeerJ. 2022 Jan 5;10:e12759. doi: 10.7717/peerj.12759 (PMC8742543; doi:10.7717/peerj.12759)

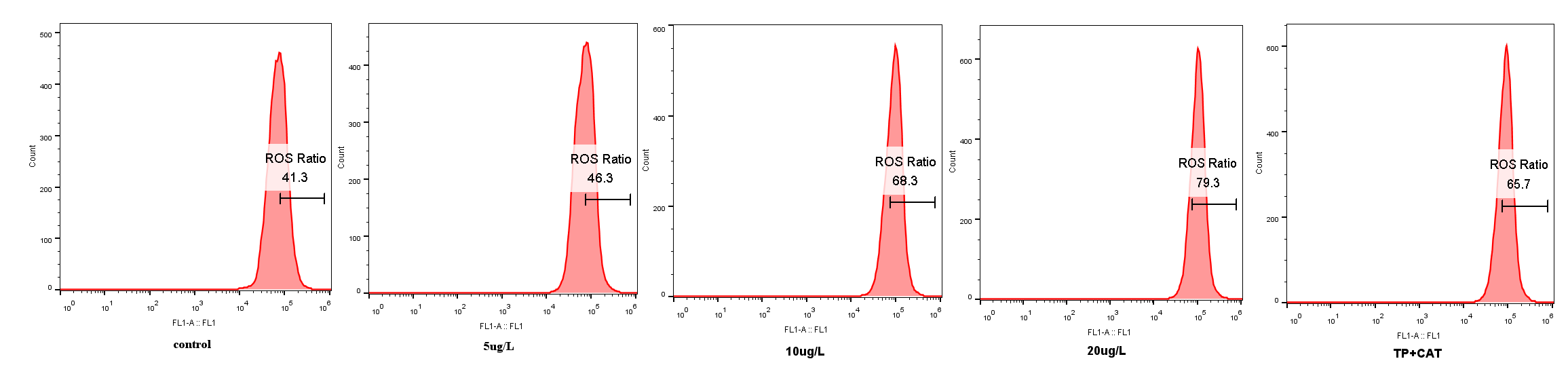

Supplement: Supplemental Information 5 — control: 5, 10, 20 ug/L [file peerj-10-12759-s005.zip › data 3-2/Flow cytometryú¿ROSú⌐/06.04ROS(K.5.10.20.20+CAT)/20210604-2-Layout.png]

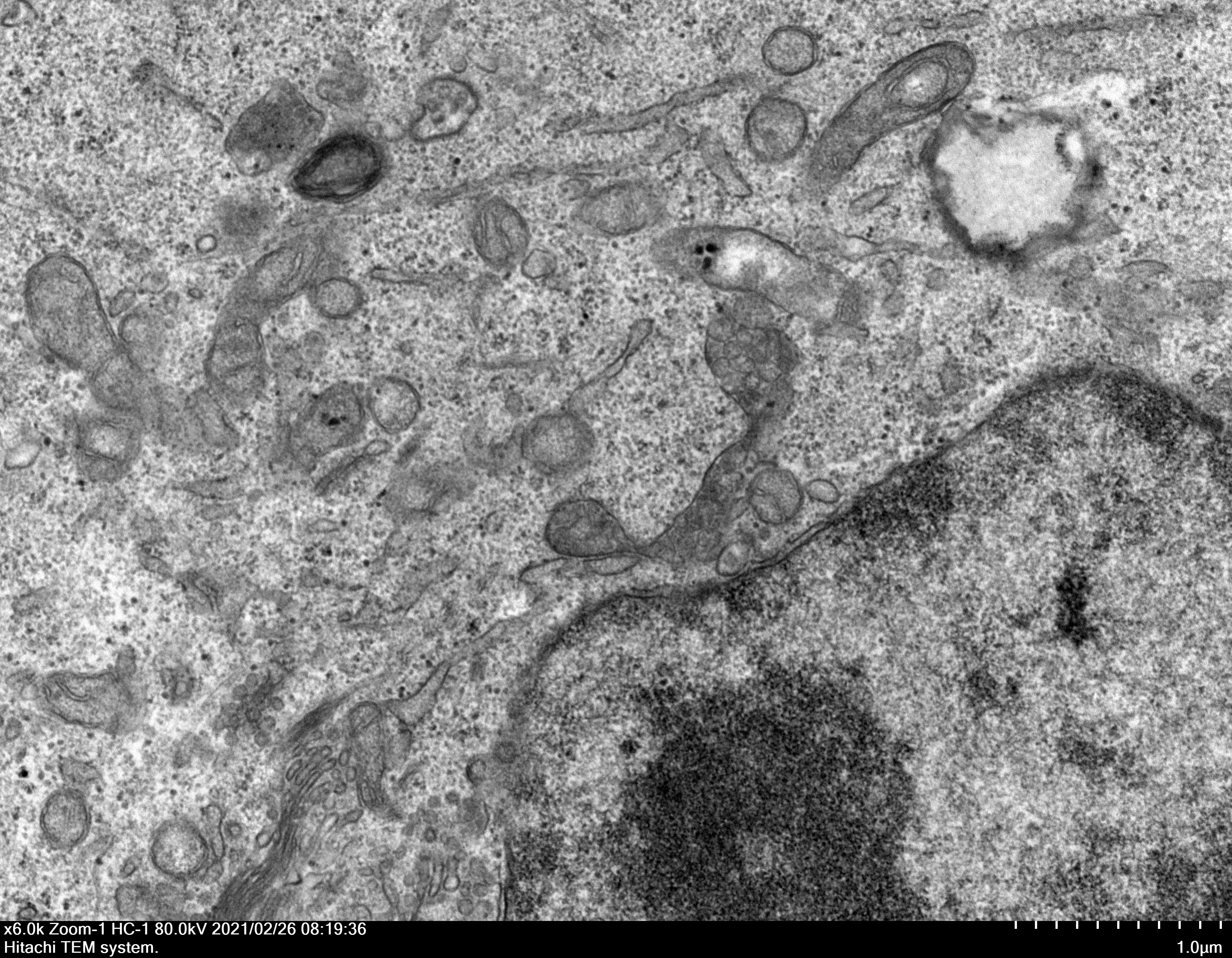

Supplement: Supplemental Information 6 — Magnification 6,000 times [file peerj-10-12759-s006.tif]

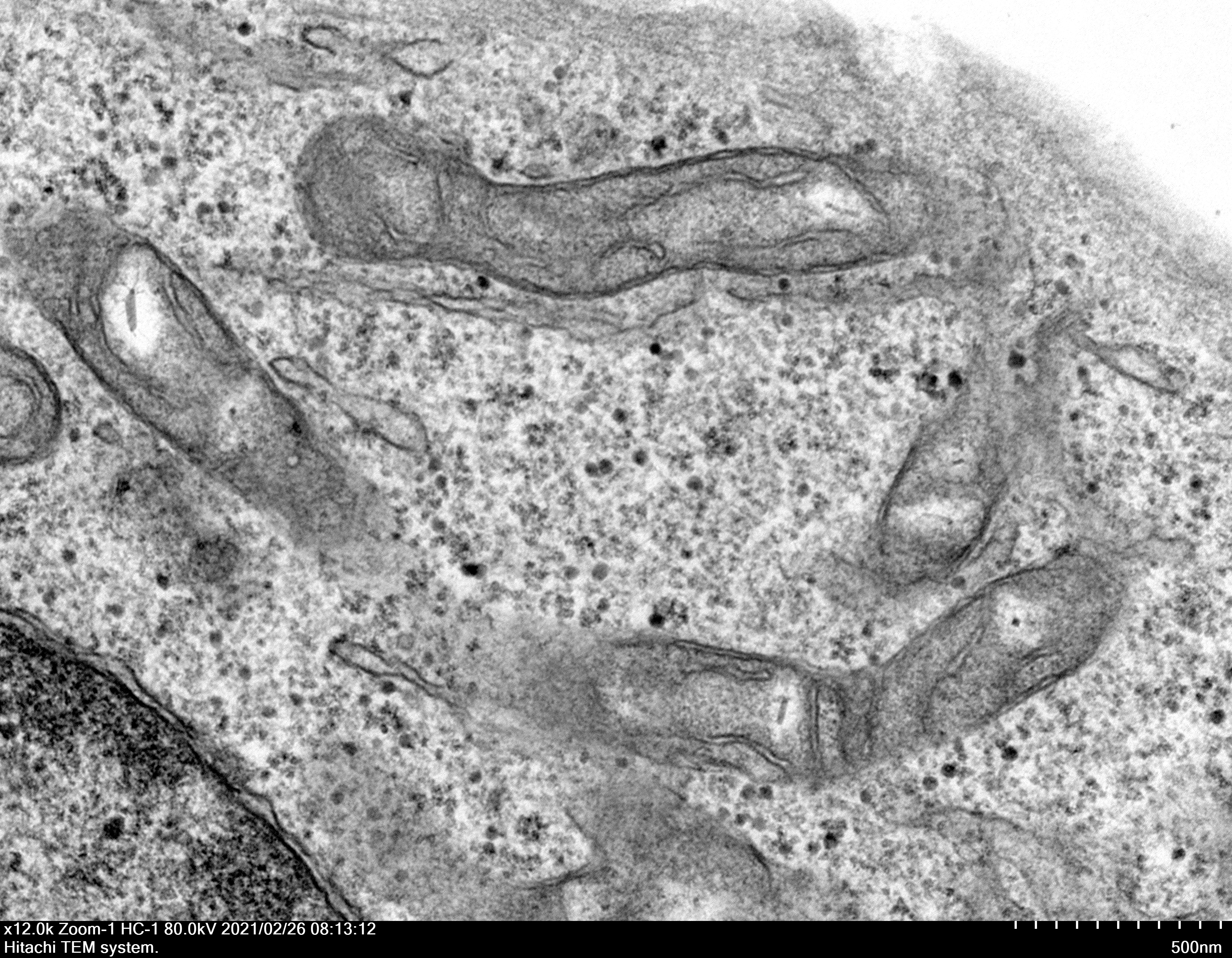

Supplement: Supplemental Information 7 — Magnification 12,000 times [file peerj-10-12759-s007.tif]

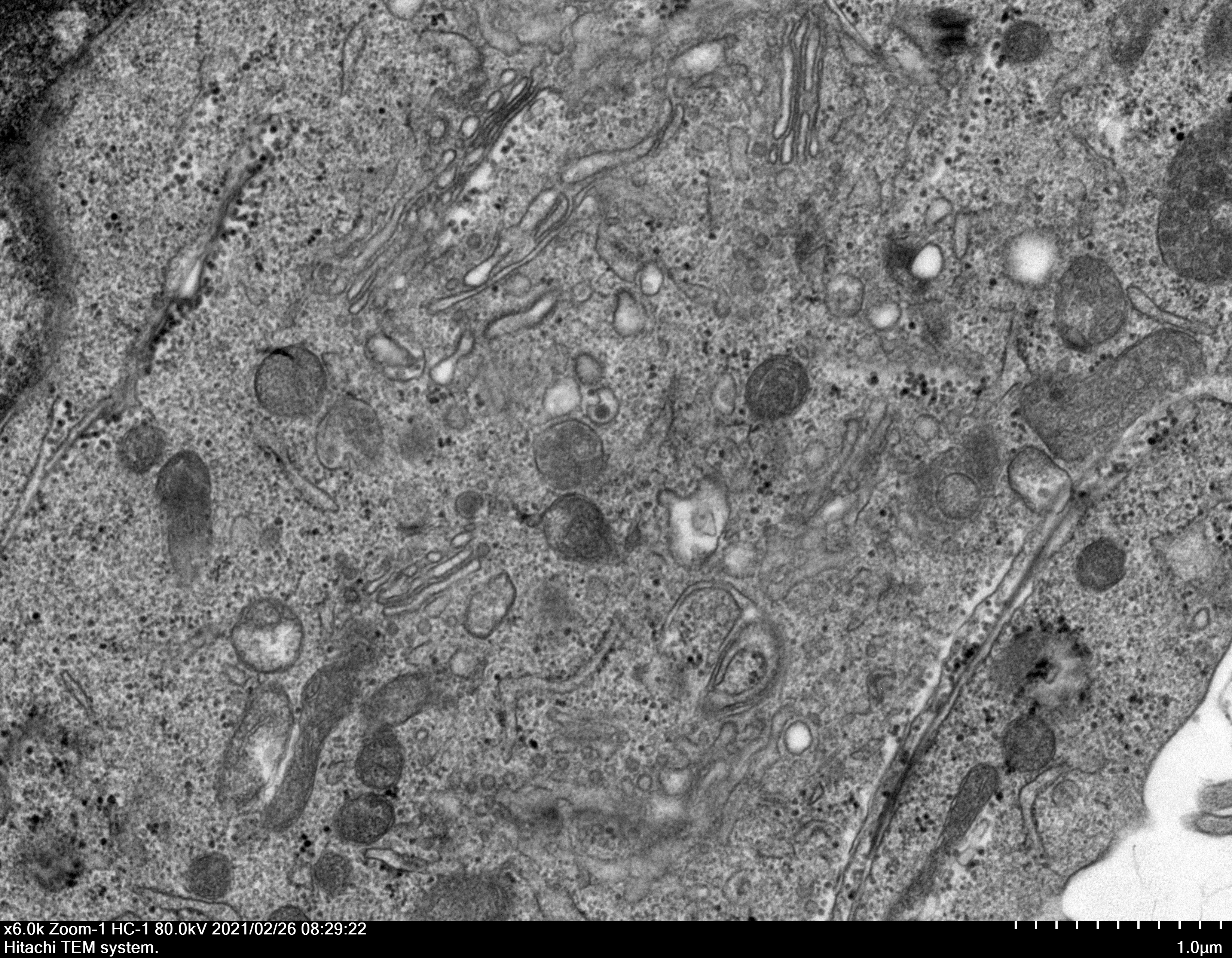

Supplement: Supplemental Information 8 — Magnification 6,000 times [file peerj-10-12759-s008.tif]

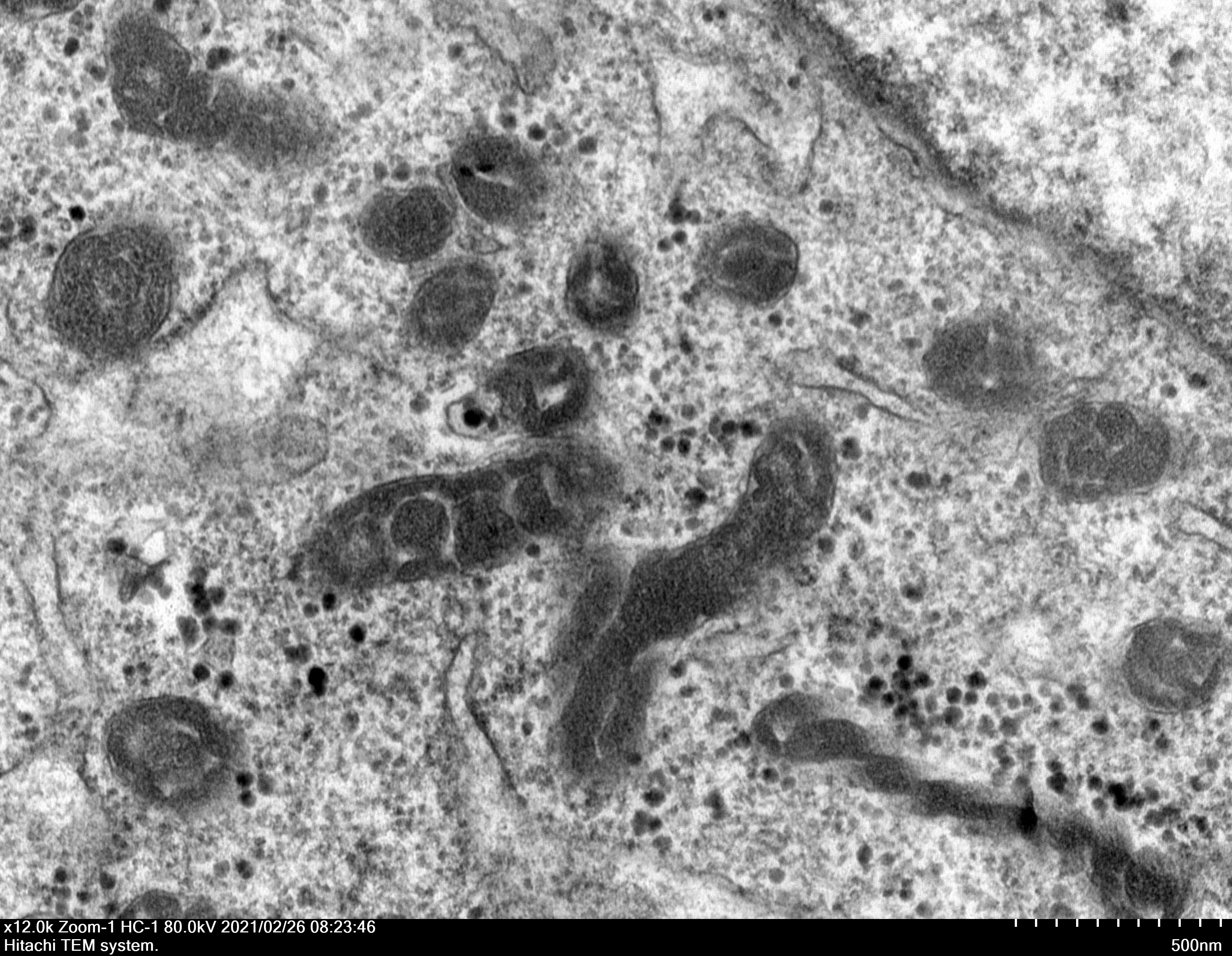

Supplement: Supplemental Information 9 — Magnification 12,000 times [file peerj-10-12759-s009.tif]

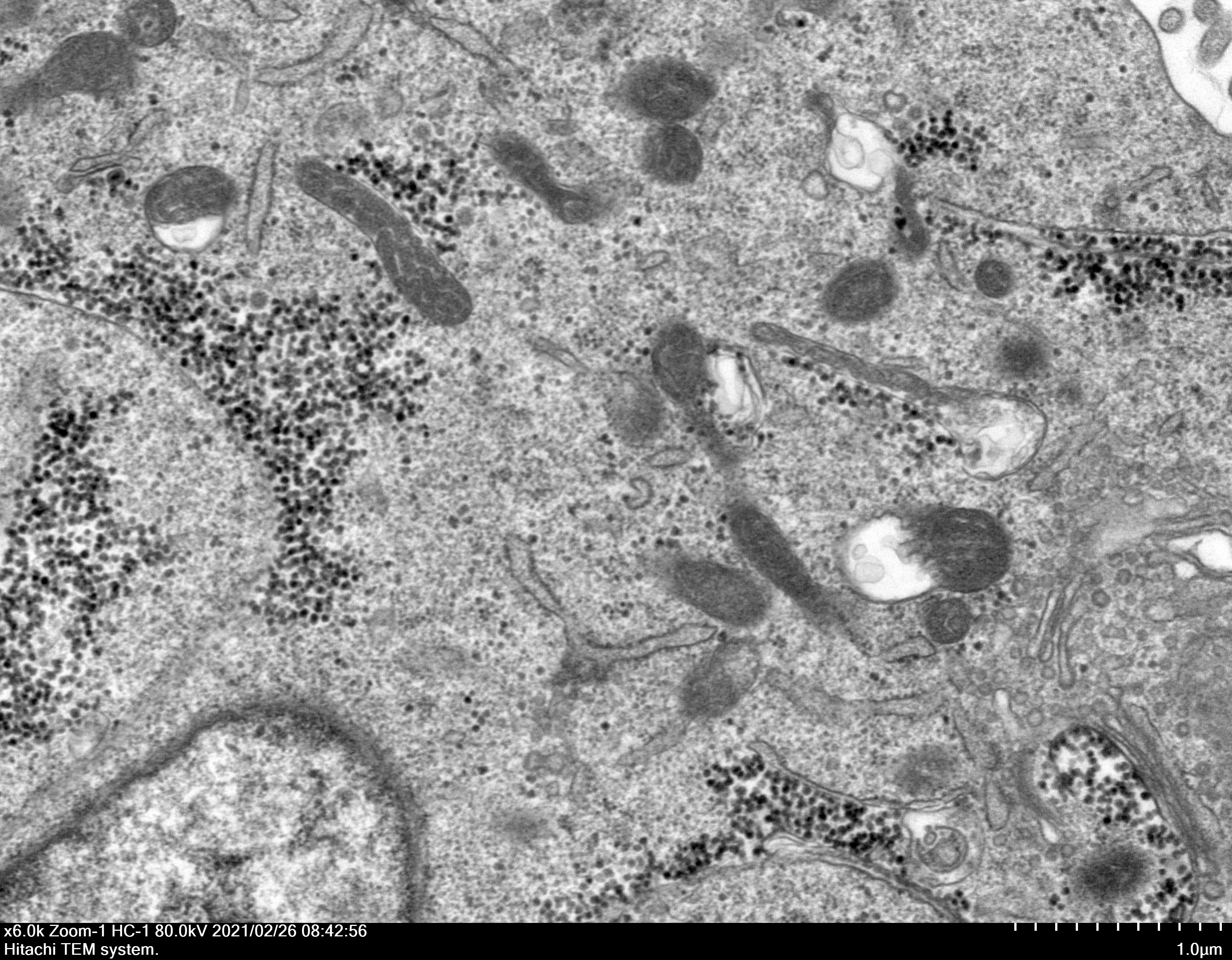

Supplement: Supplemental Information 10 — Magnification 6,000 times [file peerj-10-12759-s010.tif]

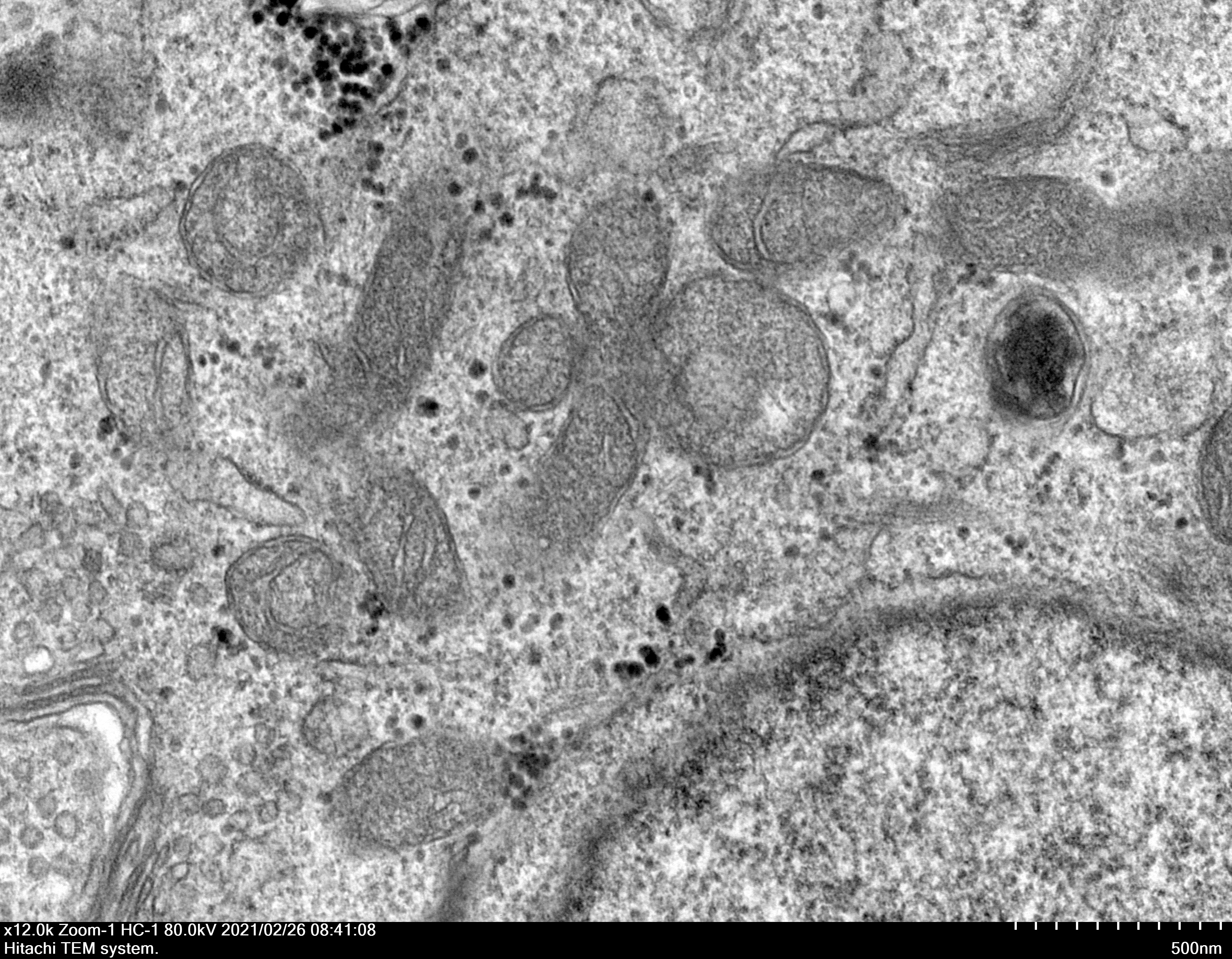

Supplement: Supplemental Information 11 — Magnification 12,000 times [file peerj-10-12759-s011.tif]
